# Longitudinal associations between well‐being, hair cortisol, and self‐reported health

**DOI:** 10.1111/aphw.12628

**Published:** 2024-12-12

**Authors:** Mario Lawes, Michael Eid

**Affiliations:** ^1^ Department of Education and Psychology Freie Universität Berlin Habelschwerdter Allee 45 Berlin Germany

**Keywords:** cortisol, eudaimonia, hair sampling, health, well‐being

## Abstract

This pre‐registered study examines the longitudinal relationships between well‐being, hair cortisol (a biomarker linked to poor health), and self‐reported health. Accumulated cortisol output over three months was determined quarterly over the course of one year using hair samples. Well‐being was assessed as a*ffective well‐being* (via experience sampling), *cognitive well‐being* (i.e., life satisfaction), and *eudaimonic well‐being* (via the Ryff Scales of Psychological Well‐Being). Self‐reported health was measured using one item on the current state of health. The longitudinal analyses allowed for disentangling initial between‐person differences from within‐person changes and were based on a large panel study of working‐age people (*N* = 726). The results indicate that hair cortisol levels were generally not associated with any of the examined well‐being facets, regardless of the level of analysis. Further, deviations from well‐being trait levels were not linked to subsequent within‐person changes in hair cortisol (and vice versa), challenging the notion that cortisol output is a key physiological pathway through which well‐being improves health. In contrast, self‐reported health was positively correlated with affective, cognitive, and eudaimonic well‐being at both the trait and within‐person levels, whereas deviations from well‐being trait levels were generally not associated with subsequent within‐person changes in self‐reported health, and vice versa.

## INTRODUCTION

Physical health and happiness are positively related to each other and essential to one's quality of life (OECD, 2013). One factor that contributes to the positive association between physical health and happiness is that poor physical health generally impairs our ability to feel happy. Interestingly, however, happiness has also been found to positively affect physical health implying a bidirectional relationship (Kushlev et al., 2020; Pressman & Cohen, 2005). Yet, while it seems evident that happiness can be advantageous for health, the mechanisms through which increased happiness translates into better health outcomes remain largely unknown (Diener et al., 2017). A promising bio‐physiological mechanism through which feeling happy might influence physical health is the output of the hormone cortisol (Steptoe, 2019). Cortisol was found to play an integral part in regulating the cardiovascular and immune systems (Kudielka & Kirschbaum, 2005) and has been repeatedly linked to happiness (Buchanan et al., 1999; Steptoe, O'Donnell, Badrick, et al., 2008). Most commonly saliva samples have been used to assess short‐term fluctuations in cortisol output (e.g., Brummett et al., 2009; Polk et al., 2005; Steptoe et al., 2005). However, cortisol measured in saliva is strongly affected by situation‐specific influences (Stalder et al., 2017). In contrast, hair cortisol concentration provides a reliable measure of accumulated cortisol output over longer periods and is increasingly recognized as the gold standard for assessing long‐term cortisol output, which has been linked to poor health (Kirschbaum et al., 2009). This pre‐registered study aims to provide an in‐depth examination of the longitudinal relationships between hair cortisol concentration and a broad set of well‐being measures that capture affective, cognitive, and eudaimonic aspects of well‐being. The hair cortisol measurements are further complemented by self‐reports of the current health status.

## WELL‐BEING AS A MULTI‐DIMENSIONAL CONSTRUCT

While pursuing happiness is a universal goal of most humans, how individuals try to achieve happiness and what they define as happiness can widely differ across individuals and situations (Heintzelman, 2018). Thus, unsurprisingly, many different conceptualizations of happiness, or more broadly well‐being, have been proposed in the scientific literature. A recent review by Andrew Steptoe (2019) highlighted the importance of distinctly measuring different facets of well‐being in health studies. Specifically, Steptoe (2019) differentiated between hedonic (or affective), evaluative (or cognitive), and eudaimonic well‐being facets. Affective well‐being is defined as experiencing positive feelings frequently and negative feelings infrequently, whereas cognitive well‐being captures how people evaluate their life overall (i.e., life satisfaction) as well as certain aspects of it (e.g., job satisfaction) (Diener, 1984; Larsen & Eid, 2008). In contrast, the eudaimonic perspective on well‐being goes back to Aristotle's Nicomachean Ethics (Aristotle, 2001) and is rooted in the idea that there is more to happiness than being satisfied with one's life and experiencing positive emotions. For example, contributing to society, engaging in meaningful tasks, and living in concordance with one's virtues can also be defining features of a happy life (Heintzelman, 2018; OECD, 2013). In the psychological literature, there are various definitions and conceptualizations of eudaimonic well‐being (for an overview see Heintzelman, 2018). The present study focuses on Carol Ryff's (1989) model of *psychological well‐being*, which defines eudaimonic well‐being using the following six dimensions: *Autonomy*, *environmental mastery*, *personal growth*, *positive relations with others*, *purpose in life*, and *self‐acceptance*.

Affective, cognitive, and eudaimonic well‐being dimensions were shown to differ in their temporal stability (Eid & Diener, 2004; Ryff et al., 2015), their relations with other variables (Lucas et al., 1996; Ryff, 1989) as well as their sensitivity toward life events (Lawes et al., 2023; Luhmann et al., 2012; Mangelsdorf et al., 2019). Considering these distinctions, it appears probable that the various well‐being dimensions are also differentially associated with physical health and the (bio‐physiological) pathways that mediate the effects of well‐being on physical health (Diener et al., 2017). However, existing studies on the relationship between well‐being and physical health have either focused on one or a few well‐being dimensions (e.g., Boehm et al., 2015) or used aggregate measures that capture a mix of different well‐being aspects (e.g., Lambiase et al., 2015). Only a few health studies have directly compared different well‐being dimensions to each other (e.g., Ryff et al., 2015; Steptoe et al., 2015; Xu & Roberts, 2010). Thus, a comprehensive investigation of the relationship between various well‐being dimensions and physical health is currently missing.

## THE RELATIONSHIP BETWEEN WELL‐BEING AND HEALTH

Various approaches have been used to study the relationship between well‐being and physical health. For example, numerous observational studies have examined the relationship between well‐being and morbidity indicating that subjective well‐being and purpose in life seem to be negatively related to morbidity (for meta‐analyses, see R. Cohen et al., 2016; Martín‐María et al., 2017). Further, subjective and eudaimonic well‐being facets were found to be positively related to physical capability and better self‐reported health in old age (Ryff et al., 2015). Moreover, well‐being facets were found to be associated with reduced risk for coronary heart disease (for a review see Boehm & Kubzansky, 2012), incident stroke (Lambiase et al., 2015), and a more favorable prognosis of various physical illnesses (for a meta‐analysis see Lamers et al., 2012). An important limitation of these studies is the lack of clarity regarding the origin of the positive relationships between well‐being and health. Generally, it is uncertain whether the well‐being facets really had a positive effect on health or whether other influences (e.g., initial differences between individuals who experience poor health outcomes and individuals who do not experience them) caused the positive associations between well‐being and health.

To make stronger inferences on whether well‐being actually *leads* to improved health researchers aimed at (i) increasing the time lag between the measurement of well‐being and the measurement of health and (ii) studying individuals who were initially highly similar to each other. A famous example in this context is the study by Danner et al. (2001), which used autobiographies of nuns to construct a measure of positive affect based on the use of positive and negative emotional words to predict the time of death. The autobiographies were written when the nuns were around 22 years old, and the mortality of the nuns was not documented until they were 75. The authors found that the estimated positive affect ratings were positively associated with the longevity of the nuns. As the nuns lived in highly similar environments this study can rule out many confounding factors. Another way to strengthen causal inference is the use of twin studies to control for genetic confounding. Sadler et al. (2011) found positive associations between subjective well‐being and mortality over a median time of 9 years in nearly 4,000 twins. These positive associations were also present in identical twins indicating that the association between well‐being and mortality cannot be entirely explained by genetic or shared environmental factors alone. Finally, the effects of well‐being on health were examined in intervention studies. For example, S. Cohen et al. (2006) administered an influenza virus to healthy participants and found that higher positive affect was associated with reduced vulnerability to the development of an upper respiratory illness. Intervention studies that aimed at directly raising well‐being (e.g., through positive psychological interventions) and studied the subsequent changes in health yielded mixed results (Diener et al., 2017; Kushlev et al., 2020). One reason for these inconclusive findings might be that positive psychological interventions or mood induction experiments often only yield small transient changes in well‐being so that sustained and meaningful health changes can generally not be expected (Heekerens & Eid, 2021; Joseph et al., 2020).

### Pathways through which well‐being might promote health

An important issue that is not well‐understood yet is through which means higher well‐being might promote health (Diener et al., 2017; Steptoe, 2019). One way seems to be through health‐related behaviors: numerous studies suggested that individuals with high levels of well‐being tend to make healthier lifestyle choices such as having a healthier diet (Grant et al., 2009), exercising more frequently (e.g., Kim et al., 2017), abstaining from smoking and other drugs (Hoyt et al., 2012), and having better sleep (e.g., Steptoe, O'Donnell, Marmot, & Wardle, 2008). These healthier behaviors then in turn have a positive effect on physical health (Pressman & Cohen, 2005; Steptoe et al., 2009).

Another potential pathway is that well‐being might trigger biological processes that are beneficial for physical health (Steptoe et al., 2009). Empirical support for the existence of such bio‐physiological pathways comes from studies showing positive associations between well‐being and the functioning of the cardiovascular and immune systems (Boehm & Kubzansky, 2012; Howell et al., 2007). A key role in this context could play the hormone cortisol as it regulates these systems (Kudielka & Kirschbaum, 2005) and has been linked to happiness (Buchanan et al., 1999; Steptoe, O'Donnell, Badrick, et al., 2008). Cortisol is the main effector of the hypothalamo‐pituitary–adrenocortical (HPA) axis, which is activated whenever a person is confronted with a stressor. Under normal circumstances, the physiological stress reaction is limited in time and adaptive as it promotes homeostasis despite the presence of stressors. In contrast, persistent cortisol output as a result of chronic stress has been shown to be associated with numerous negative health outcomes such as cardiovascular disease, obesity, and type 2 diabetes (Stalder et al., 2017).

Several field and laboratory studies have examined the relationship between well‐being and cortisol. These studies generally found that higher well‐being levels were associated with “healthier” patterns of short‐term cortisol output. Specifically, positive affect was found to be associated with (i) lower saliva cortisol levels (Steptoe et al., 2005; Steptoe, O'Donnell, Badrick, et al., 2008; for a contrasting finding, see Jacobs et al., 2007), (ii) steeper declines in saliva cortisol over the day (Polk et al., 2005), and (iii) lower saliva cortisol after awakening (Brummett et al., 2009). Most existing studies on well‐being and cortisol output, however, have two important limitations. First, they generally focus on positive affect so that not much is known about how cognitive or eudaimonic well‐being facets are related to cortisol output. Second, most existing studies used saliva samples to measure cortisol. Whereas saliva cortisol is excellent to assess short‐term changes in cortisol output, is not well‐suited to measure long‐term cortisol output, which is more strongly related to poor health (Stalder et al., 2017). To measure cortisol output over extended periods, hair cortisol analysis has become the gold standard method (Kirschbaum et al., 2009). As human hair grows at a fairly predictable rate of 1 cm per month (Loussouarn et al., 2005), aggregated cortisol levels over multiple months can be examined retrospectively in hair samples. The accumulated cortisol output in hair over one month was shown to be highly correlated with the 30‐day average across three daily saliva samples within the same period (Short et al., 2016). Despite these advantages of hair cortisol analysis, we are unaware of studies relating hair cortisol concentration to well‐being measures.

## THE PRESENT STUDY

This pre‐registered study aims to provide an in‐depth examination of the longitudinal relationships between health and well‐being. Further, it follows up on previous findings suggesting that changes in cortisol output could be an important bio‐physiological pathway through which well‐being manifests itself as better health. This study extends the existing literature in three important ways. First, it uses hair samples to determine the accumulated cortisol output over three months, a biomarker that is strongly linked to poor health. Second, it utilizes a broad set of well‐being measures that capture affective, cognitive, and eudaimonic aspects of well‐being and complements the hair cortisol measures with self‐reports on the current health status. Third, the analyses are based on a large longitudinal sample of working‐age people with five quarterly assessments spanning over one year and allow for disentangling initial between‐person differences from within‐person changes. Based on the literature discussed above, four directional hypotheses H1 – H4 were derived and pre‐registered^1^ (see https://osf.io/dg8u7). H1 states that individuals with higher dispositional well‐being levels will generally report better health and have lower levels of hair cortisol. H2 states that within‐person fluctuations of well‐being and self‐reported health are positively correlated at a given measurement wave, while concurrent within‐person fluctuations of well‐being and hair cortisol concentration are negatively correlated. H3 states that within‐person fluctuations in individuals' well‐being will positively predict subsequent fluctuations in self‐reported health and negatively predict subsequent fluctuations in hair cortisol levels. H4 states that within‐person fluctuations in individuals' self‐reported health will positively predict subsequent fluctuations in well‐being levels, while fluctuations in individuals' hair cortisol levels will negatively predict subsequent fluctuations in well‐being.

## METHODS

### Data

The study is based on data from the German Job Search Panel (GJSP; Hetschko et al., 2022), a monthly app‐based panel study with five quarterly hair collection waves. To recruit its participants, the GJSP exploited that German employees are required to register as jobseekers at least three months prior to their expected job loss. Based on these job search registrations, individuals who were still employed but expected to lose their jobs were identified. GJSP participants were recruited as two cohorts, one before and one during the COVID‐19 pandemic. For the first cohort, 79,711 jobseekers who were likely to be affected by mass layoffs and 48,126 jobseekers who were likely to lose their jobs due to other reasons were identified between November 2017 and May 2019. For the second cohort, 42,340 jobseekers all of whom were likely to be affected by mass layoffs were identified between July 2020 and February 2021. Identified individuals were contacted via letter or email (Lawes, Hetschko, Sakshaug, & Grießemer, 2022). A total of 6,591 individuals (*N*_cohort1_ = 4,700, *N*_cohort2_ = 1,891) started filling out the entry survey, which was used to determine the eligibility for the study. Individuals who had already entered unemployment (*N*_cohort1_ = 1,446, *N*_cohort2_ = 711) or who had been employed for less than six months (*N*_cohort1_ = 216, *N*_cohort2_ = 99) were excluded. Further, one‐third of all individuals of the first cohort (*N* = 950) were randomly excluded after the entry survey to investigate the role of survey participation on employment‐related outcomes (see Stephan et al., 2024). Individuals were also excluded when they did not submit the entry survey (*N*_cohort1_ = 246, *N*_cohort2_ = 121) or when they did not participate in the GJSP after the entry survey (*N*_cohort1_ = 302, *N*_cohort2_ = 51). Accordingly, 2,449 (*N*_cohort1_ = 1,540, *N*_cohort2_ = 909) individuals were included in the final sample. The present study is based on *N* = 726 individuals who sent in at least one valid hair sample^2^ throughout the study (*N*_cohort1_ = 449, *N*_cohort2_ = 277). Table 1 summarizes the key characteristics of the analyzed sample (see Table S1 in the supplementary materials to find this information detailed separately by cohort).

**TABLE 1:** Characteristics of participants at baseline (*N* = 726).

	*N*	%
*Gender*		
Female	487	67.1
Male	238	32.8
Other	1	0.1
*College degree*	405	56.1
*Married*	323	45.0
*Hair color*		
Blond	343	50.0
Brown	295	43.0
Black	15	2.2
Red	32	4.7
Grey/white	1	0.1
*Dyed or bleached hair*	198	28.7
*Tinted hair*	71	9.8

*Note*: Participants were on average 39.43 years old (*SD* = 10.16), had a mean BMI of 25.39 (*SD* = 5.46), and washed their hair an average of 3.71 times a week (*SD* = 2). These characteristics are further detailed separately by cohort in Table S1 of the supplementary materials.

#### Procedure

Over up to 25 months, participants received monthly questionnaires assessing a wide range of information via a specifically developed smartphone app (for details see Hetschko et al., 2022). The parallel collection of hair samples ran on a quarterly basis from the beginning of study participation for up to one year. Previous analyses based on the first GJSP cohort showed that the effects of selective participation in the hair collection were small (Lawes et al., 2024). The study protocol was approved on Dec 13, 2017, by the ethics committee of the Department of Education and Psychology at Freie Universität Berlin and informed consent was obtained from all respondents at the start of the entry survey.

### Measures

The wordings of all utilized questionnaire items are presented in Materials S1 in the supplementary materials.

#### Momentary happiness

The experience sampling method (ESM; Hektner et al., 2007) was used to assess momentary happiness as a measure of affective well‐being. At the last day of each monthly survey wave, participants received six short ESM questionnaires at randomly chosen times throughout the day between 8 am and 9 pm. At each ESM episode, individuals rated the statement “In the moment I feel happy” on a 5‐point rating scale ranging from *not at all* (1) to *very much* (5) (see Multidimensional Mood State Questionnaire; Steyer et al., 1994). To obtain an indicator of momentary happiness, the responses to this item were averaged across all submitted ESM episodes of a given survey day (for details see Lawes et al., 2023). These monthly scores were then transformed into percent of maximum possible scores (POMP; P. Cohen et al., 1999). POMP scores range from 0 to 100 and can be interpreted in terms of percentage points, thus serving as an easily interpretable measure of effect size.

#### Life satisfaction

Life satisfaction was measured with the Satisfaction With Life Scale (SWLS; Diener et al., 1985) as a measure of cognitive well‐being at each monthly wave of the GJSP. Participants responded to the five SWLS items using a 7‐point rating scale ranging from *strongly disagree* (1) to *strongly agree* (7). The first three items of the SWLS were used to compute scale scores, which were then transformed into POMP scores. Items 4 and 5 of the SWLS were not utilized as they refer to longer time periods.

#### Eudaimonic well‐being

The six dimensions of eudaimonic well‐being (i.e., self‐acceptance, positive relations with others, autonomy, environmental mastery, personal growth, and purpose in life) were assessed monthly using an adapted 24‐item version of a German translation of the Ryff‐Scale for Psychological Well‐Being (Risch et al., 2005; Ryff, 1989). Each of the six eudaimonic well‐being dimensions was assessed with four items using a 4‐point rating scale ranging from *completely disagree* (1) to *completely agree* (4). For each dimension, monthly scale scores were computed and transformed into POMP scores.

#### Hair cortisol concentration

On the seventh measurement day of the monthly survey waves 1, 4, 7, 10, and 13, individuals were asked via the survey app whether they were willing to send in a hair sample to have their hair cortisol concentration determined. In the following, we will label these five hair collection waves Q1 – Q5 to signal that they refer to quarterly measurements. Respondents who indicated that (i) their hair was shorter than 2 cm or (ii) they took cortisone‐based medication were excluded from the hair collection. Further, individuals who were not willing or eligible to participate in the first hair collection wave were excluded from later collection waves. Hair samples were self‐collected by the respondents. For this purpose, eligible respondents received hair collection kits via mail. These kits contained detailed instructions for hair removal, loops to fixate the hair strands, aluminum foil for dry and dark shipping, a prepaid return envelope, and a paper‐pencil questionnaire to assess factors that may confound hair cortisol values, such as hair color, frequency of hair washing and cortisone‐based medication.^3^ Respondents were asked to send in three hair strands of 3 mm diameter within 10 days after receiving the collection kit (for details on the hair collection see Lawes et al., 2024). Previous research has emphasized that self‐collection of hair samples is a cost‐effective procedure that produces results very similar to those of professionally collected samples (Enge et al., 2020).

The 3 cm hair segments closest to the scalp were analyzed in batches over several years by the bio laboratory “Dresden Lab Service” using immunoassays, following the protocol by Davenport et al. (2006). The 3 cm hair segments provide a measure of cumulative cortisol exposure over the past three months. This three‐month period was chosen because it is long enough to assess accumulated cortisol levels while being short enough to study changes over time. Additionally, this timeframe has been used in most existing hair cortisol studies (see Schaafsma et al., 2021), allowing for comparability with previous results. The Dresden Lab Service ensured that the intraassay and interassay coefficient of variance of the utilized assays was below 8%. As a quality control measure, 10% of the hair samples were further analyzed using liquid chromatography–tandem mass spectrometry (LC–MS/MS). Reassuringly, the cortisol concentrations obtained from both methods correlated between 0.95 and 0.999 across the five hair collection waves. Since hair cortisol data are typically not normally distributed and the utilized statistical analyses are sensitive to outliers, data points below the 5th percentile and above the 95th percentile were replaced with the values at those percentiles. This process, known as winsorization, helps to mitigate the influence of extreme values. Further, the winsorized hair cortisol concentrations were log‐transformed separately for each collection wave (analogous to Lawes, Hetschko, Schöb, et al., 2022).

#### Self‐reported health

Self‐reported health was measured monthly using the item “How would you rate your current state of health?” (Engstler et al., 2013). Individuals responded on a 5‐point rating scale ranging from *very bad* (1) to *very good* (5). The responses to this item were then also transformed into POMP scores.

### Data aggregation

The monthly data of the well‐being measures and the self‐reported health item were aggregated across the three measurement occasions prior to the quarterly hair collection occasions (analogous to Lawes, Hetschko, Schöb, et al., 2022). This way the aggregated scale scores correspond to the same timeframe as the hair cortisol data. For the first measurement occasion, the scale scores of the first measurement occasion were used. The analyses then relied on the obtained five measurement occasions of hair cortisol, (aggregated) self‐rated health, and (aggregated) affective, cognitive, and eudiamonic well‐being.

### Analytical model

**FIGURE 1:**
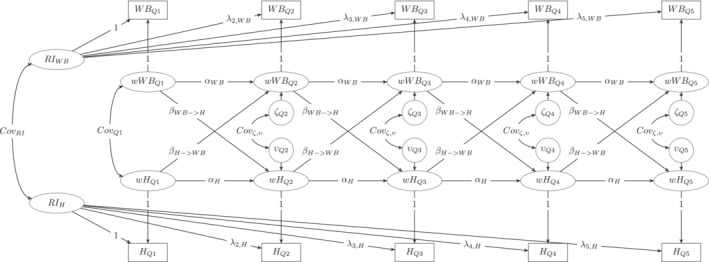
Path model of the analysis model. Note. *WB*_*Q*1_ – *WB*_*Q*5_ represent the observed (aggregated) scale scores of the well‐being dimensions at the five measurement occasions Q1 – Q5. Analogously, *H*_*Q1*_ – *H*_*Q5*_ are the five quarterly health measurements (i.e., hair cortisol and self‐rated health). *RI*_*WB*_ and *RI*_*H*_ are the random‐intercept variables that capture trait differences in terms of the well‐being and health facets. *wWB*_*Q1*_ ‐ *wWB*_*Q*5_ and *wH*_*Q*1_ ‐ *wH*_*Q*5_ capture occasion‐specific deviations of a person's observed score from their expected score based on the random intercepts. $\alpha_{\mathrm{WB}}$ and $\alpha_{H}$ represent autoregressive effects and $\beta_{\mathrm{WB}\to H}$ and $\beta_{H\to \mathrm{WB}}$ cross‐lagged effects at the within‐person level. The autoregressive and cross‐lagged effects were both set equal across time in all analysis models. $\zeta_{Q2}$ ‐ $\zeta_{Q5}$and $\nu_{Q2}$ ‐ $\nu_{Q5}$ are occasion‐specific residual variables, the occasion‐specific covariances of these variables were also set equal across time. λ's represent factor loadings of the random intercept variables. These could be restricted to 1 for some models and were freely estimated for others (see Table S3a, S3b, and S4 in the supplementary materials).

Sixteen separate bi‐variate random intercept cross‐lagged panel models (RI‐CLPM; Hamaker et al., 2015) were fitted for each combination of the eight well‐being facets (i.e., life satisfaction, momentary happiness, six facets of eudaimonic well‐being) and the two health measures (i.e., hair cortisol and self‐reported health). RI‐CLPMs are well‐suited for the present study as they separate stable between‐person differences from within‐person changes occurring over time, so that temporal dynamics over time and stable interindividual differences can be examined separately. Potential confounders due to time‐invariant factors (e.g., biological gender, age) are captured by the random intercepts, thereby preventing them from biasing the estimation of within‐person effects. A path diagram of the utilized model is depicted in Figure 1. *WB*_*Q*1_ – *WB*_*Q*5_ represent the observed (aggregated) scale scores of the well‐being dimensions at the five measurement occasions Q1 – Q5. Analogously, *H*_*Q*1_ – *H*_*Q*5_ are the five quarterly health measurements (i.e., hair cortisol or self‐rated health). *RI*_*WB*_ and *RI*_*H*_ are the random‐intercept variables that capture stable trait differences in terms of the particular well‐being and health indicator at the first measurement occasion (for a detailed discussion see Eid et al., 2017). The correlation between *RI*_*WB*_ and *RI*_*H*_ capture the trait level correlations of the well‐being and health measures, which are central to H1. *wWB*_*Q*1_ ‐ *wWB*_*Q*5_ and *wH*_*Q*1_ ‐ *wH*_*Q*5_ capture occasion‐specific deviations of a person's observed score from their expected score based on their initial trait levels. For example, a positive value on *wWB*_*Q*3_ would indicate that a person reported higher well‐being at Q3 than what would have been expected due to their trait levels at Q1. The autoregressive effects $\alpha_{\mathrm{WB}}$ and $\alpha_{H}$ model transfer effects at the within‐person level (i.e., deviations at the previous occasion can influence the deviations at the next occasion). The cross‐lagged effects $\beta_{\mathrm{WB}\to H}$ and $\beta_{H\to \mathrm{WB}}$ allow for the well‐being deviations at occasion *t* to predict the deviation in health at *t*+1 (i.e., in the subsequent three months), and vice versa. These cross‐lagged effects address H3 and H4. Lastly, the occasion‐specific residuals $\zeta_{Q2}$ ‐ $\zeta_{Q5}$and $\nu_{Q2}$ ‐ $\nu_{Q5}$ capture the within‐person deviations at Q2 – Q5 that cannot be explained by the autoregressive and cross‐lagged effects. Correlations between *wWB*_*Q*1_ and *wH*_*Q*1_ as well as between $\zeta_{t}$ and $\nu_{t}$ were permitted, the latter being central to examine H2.

As depicted in Figure 1, we aimed at restricting the within‐person processes (i.e., auto‐regressive and cross‐lagged effects as well as the [co‐]variances of the occasion‐specific residuals) to be constant over time. Further, we aimed to restrict the factor loadings of the random intercept variables to be 1. To examine, whether these restrictions are justified, we compared the model fit of the restricted model versions to (i) models in which there were no restrictions and (ii) models in which only the within‐process was assumed to be constant over time but the factor loadings of the random intercept variables were freely estimated (expect for the first loading, which was set to 1 in order to identify the model). For each of the 16 models, the most appropriate model was then selected based on the chi‐squared difference test and the BIC. When the chi‐squared difference test and the BIC suggested different models, we chose the model with the smallest BIC to select the most parsimonious model that still fits the data well.

All hypotheses were tested using a significance level of α = .05 and one‐sided tests. H1 was examined by testing the correlations between the random intercept variables. H2 was examined by testing the correlations between the occasion‐specific residual variables. H3 and H4 were examined by testing the cross‐lagged effects. Given the large number of effects tested, we corrected the *p*‐values using the Benjamini–Hochberg procedure to control for a false discovery rate of α = .05 (Benjamini & Hochberg, 1995). To compute the corrected *p*‐values, we separately applied the R function “p.adjust” to the 16 uncorrected *p*‐values resulting from the 16 combinations of well‐being facets and health measures (i.e., hair cortisol and self‐rated health) for each of the hypotheses.

### Computational procedure and data availability

All models were fitted using lavaan (version 0.6–16; Rosseel, 2012) in R (version 4.3.1; R Core Team, 2017). We used the robust full information maximum likelihood estimator to handle missing data and to account for the nonnormal distribution of the indicators while utilizing all available information. The analysis scripts are available at the online repository of this study (https://osf.io/9xbd5/). The data are available for research purposes upon request and can be obtained by contacting the corresponding author.

## RESULTS

### Descriptive results

Table S2 in the supplementary materials depicts the means, standard deviations, and sample sizes for all measures on the five quarterly occasions. Figure S1 in the supplementary materials illustrates the bi‐variate product‐moment correlations between all measures over the five occasions Q1 – Q5.

### Model comparisons

Tables S3a and S3b in the supplementary materials present the model fit for all models. Five of the 40 fitted models (12.5%) did not converge or yielded negative variances or covariances. In all these models there were no restrictions on the within‐process or the factor loadings of the random intercept variables. Based on the BIC, the within‐person process (i.e., the autoregressive and cross‐lagged effects, [co‐]variances of occasion‐specific residuals) could be restricted to be equal over time for all models, whereas the factor loadings on the random‐intercept variables had to be freely estimated in some models and could be fixed across time in others. Table S4 in the supplementary materials depicts the factor loadings for all selected analysis models.

### Correlations of random intercept variables (H1)

Table 2 (upper section) depicts the correlations of the random‐intercept variables. These correlations address H1 as they capture the linear relationships between the trait levels of the well‐being facets with hair cortisol and self‐reported health. The trait level correlations between the well‐being facets and hair cortisol concentration ranged from −.08 to .10 with none of the correlations being significantly smaller than zero after applying the Benjamini‐Hochberg correction. The trait level correlations between the well‐being facets and self‐rated health ranged from .27 to .66 with all correlations being significantly greater than zero after applying the Benjamini‐Hochberg correction (one‐sided *p*‐values < .001).

**TABLE 2:** Product–moment‐correlations of random‐intercept variables (trait level) and occasion‐specific residuals (within‐person level).

	Hair cortisol			Self‐reported health		
	Estimate	90%‐CI	*p* (corrected)	Estimate	90%‐CI	*p* (corrected)
*Trait level*						
Momentary happiness	.047	[−.037; .132]	.389	**.572**	[.514; .63]	< .001
Life satisfaction	−.034	[−.113; .045]	.345	**.660**	[.607; .712]	< .001
Positive relations with others	.046	[−.03; .122]	.389	**.426**	[.365; .487]	< .001
Autonomy	.095	[.022; .169]	.483	**.273**	[.201; .344]	< .001
Self‐acceptance	.036	[−.041; .113]	.374	**.503**	[.443; .562]	< .001
Psychological growth	−.085	[−.165; ‐.004]	.073	**.413**	[.347; .479]	< .001
Sense of purpose	−.041	[−.119; .036]	.302	**.468**	[.406; .529]	< .001
Environmental mastery	.075	[−.001; .151]	.477	**.502**	[.442; .562]	< .001
*Within‐person level*						
Momentary happiness	.045	[−.019; .109]	.403	**.187**	[.137; .237]	< .001
Life satisfaction	.026	[−.037; .089]	.337	**.159**	[.108; .21]	< .001
Positive relations with others	−.023	[−.091; .045]	.359	**.096**	[.045; .147]	.003
Autonomy	−.049	[−.112; .014]	.18	.055	[.007; .102]	.057
Self‐acceptance	.024	[−.038; .085]	.337	**.167**	[.122; .211]	< .001
Psychological growth	.072	[.002; .141]	.456	**.129**	[.08; .179]	< .001
Sense of purpose	−.016	[−.083; .052]	.402	**.069**	[.018; .119]	.031
Environmental mastery	−.029	[−.094; .036]	.337	**.152**	[.105; .2]	< .001

*Note*: The 90%‐confidence intervals (CI) were computed since one‐sided tests were used to test the correlations against zero at α = .05; the *p*‐values correspond to one‐sided *p*‐values that were corrected using the Benjamini‐Hochberg procedure, estimates with corrected *p*‐values smaller than .05 are shown in bold. The sample size for all analyses is *N* = 726.

### Correlations of occasion‐specific residual variables (H2)

Table 2 (lower section) depicts the correlations of the occasion‐specific residual variables. These correlations address H2 as they capture the linear relationships between deviations from the well‐being trait levels and concurrent deviations from the hair cortisol trait levels and self‐reported health trait levels. The within‐person correlations between the well‐being facets and hair cortisol ranged from −.05 to .07 with none of the correlations being significantly smaller than zero after applying the Benjamini‐Hochberg correction. The within‐person correlations between the well‐being facets and self‐rated health ranged from .05 to .19 with all correlations being significantly greater than zero (one‐sided *p*‐values < .05) after applying the Benjamini‐Hochberg correction, except for the correlation between autonomy and self‐rated health (ρ = .06, corrected one‐sided *p*‐value = .057).

### Cross‐lagged‐effects

**FIGURE 2:**
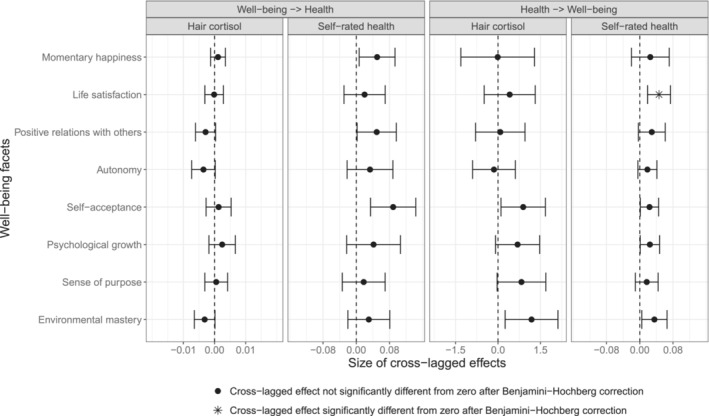
Cross‐lagged effects. Note. The points depict the cross‐lagged effects, the whiskers denote the 90%‐confidence interval (i.e., one‐sided tests). Note that the scaling of the x‐axis differs between the plots, this is due to the different metrics in which the variables were analyzed. The sample size for all analyses is *N* = 726. The numeric values underlying this figure are presented in tables S5a and S5b in the supplementary materials.

Figure 2 illustrates the estimated cross‐lagged effects with their corresponding 90%‐confidence intervals, which address H3 and H4. The 90%‐confidence intervals were computed since a one‐sided test was used to test the effect against zero at α = .05. It is important to note that the absolute size of the coefficients cannot be easily compared to each other as hair cortisol concentration has a vastly different metric than the well‐being facets and self‐reported health. Specifically, the hair cortisol levels are log‐transformed cortisol levels (in pg/mg hair) that range from 0.19 to 2.97, whereas the well‐being and self‐reported health scores range from 0 to 100 (i.e., POMP scores). The autoregressive effects of the models are presented in Table S6 in the supplementary materials; they are not discussed in the following as they are not of substantive interest in the present study.

#### Cross‐lagged effects of well‐being on physical health (H3)

The cross‐lagged effects of the trait level deviations in the well‐being facets predicting the subsequent trait level deviations in hair cortisol within the following three months ranged from −0.004 to 0.002 with none of them being significantly smaller than zero. The trait level deviations in the well‐being facets predicting subsequent trait level deviations in self‐reported health within the following three months ranged from 0.02 to 0.09 with none of them being significantly greater than zero.

#### Cross‐lagged effects of physical health on well‐being (H4)

The cross‐lagged effects of the trait level deviations in hair cortisol predicting subsequent trait level deviations in the well‐being facets within the following three months ranged from −0.14 to 1.18 with none of them being significantly smaller than zero. The trait level deviations in self‐reported health predicting subsequent trait level deviations in the well‐being facets within the following three months were all positive and ranged from 0.02 to 0.05. However, only the cross‐lagged effects of self‐reported health predicting life satisfaction within the next three months was significantly greater than zero (corrected one‐sided *p*‐value = .046).

### Sensitivity analyses

Besides the pre‐registered analyses, we conducted two sensitivity analyses to probe the robustness of the results. First, we re‐ran all analyses separately for the two GJSP cohorts in order to examine whether the COVID‐19 pandemic and its associated psychological and physiological challenges might affect the results (see e.g., Marcil et al., 2022). The results were highly consistent across the two cohorts and closely resembled those of the full sample (see Table S7 and Figure S2 in the supplementary materials). The only noteworthy difference was that in cohort 2 (i.e., during the COVID‐19 pandemic) the trait level correlation between life satisfaction and hair cortisol concentration was significantly smaller than zero (ρ = −.16, corrected one‐sided *p*‐value = .029). Further, when the two cohorts were analyzed separately the cross‐lagged effects of self‐reported health predicting subsequent life satisfaction were no longer significantly greater than zero. As a second sensitivity analysis, we re‐ran the analysis with a subsample of continuously employed individuals (*N* = 224) in order to examine whether employment‐related uncertainty might confound the results. However, also the results of the second sensitivity analysis were highly similar to those based on the full sample (see Table S8 and Figure S3 in the supplementary materials). The only exception was that the trait level correlation of hair cortisol and sense of purpose was significantly smaller than zero in this sample (ρ = −.18; corrected one‐sided *p*‐value = .027).

## DISCUSSION

The present study examined the longitudinal relationships between affective, cognitive, and eudaimonic well‐being facets with (i) hair cortisol concentration, a biomarker that is linked to poor health, and (ii) self‐reported health. Five quarterly measurements were used to examine the relationships at the trait level (i.e., between‐person differences) and at the level of occasion‐specific deviations from the trait levels (i.e., within‐person fluctuations). The first two hypotheses tested correlations of the well‐being facets with hair cortisol levels and self‐rated health at the trait and within‐person levels. Hypotheses 3 and 4 tested whether within‐person fluctuation in well‐being predicted subsequent within‐person fluctuations in hair cortisol output and self‐rated health, and vice versa.

### Correlations of health and well‐being (H1 and H2)

Contrary to the first hypothesis, the results indicate that none of the well‐being facets were correlated with hair cortisol concertation at the trait level. In contrast, self‐reported health was positively correlated at the trait level with all well‐being facets with moderate to large correlations according to Jacob Cohen (1992). The trait level correlations of self‐reported health were particularly large with life satisfaction (*r* = .66) and momentary happiness (*r* = .57), whereas considerably smaller for autonomy (*r* = .27). Thus, the analyses support H1 for self‐reported health but not for hair cortisol concentration.

The second hypothesis stated that at a given occasion, within‐person deviations from the well‐being traits levels would be negatively correlated with within‐person deviations from the hair cortisol concertation trait levels and positively correlated with within‐person deviations from the self‐reported health traits levels. Analogously to the results at the trait level, this hypothesis was only supported for self‐reported health but not for hair cortisol concentration. The largest correlations of self‐reported health at the within‐person level were found with momentary happiness (*r* = .19) and self‐acceptance (*r* = .17), whereas autonomy had (again) the smallest correlation with self‐reported health (*r* = .06, *ns*.).

Overall, the correlational analyses therefore suggest that hair cortisol levels are not linearly related to any of the examined well‐being facets regardless of the level of analysis. One explanation for the absence of these associations might be that the cortisol system seems to be especially sensitive to uncontrollable situations, unfamiliar challenges, and social‐evaluative threats (Dickerson & Kemeny, 2004; Lawes, Hetschko, Schöb, et al., 2022; Mayer et al., 2018). In contrast, determinants of well‐being have been shown to be highly person‐ and situation‐specific (Diener et al., 1999). This explanation is in line with several other studies that also found no correlation between hair cortisol concentration and a variety of different psychological constructs, including perceived stress and depressiveness (Schaafsma et al., 2021; Stalder et al., 2017). Further research is needed to more directly examine this explanation and to investigate how methodological issues, such as the timeframe of the study and the method of hair sample collection (self‐collection vs. professional collection), might affect the results. In contrast, self‐rated health was positively correlated with almost all examined well‐being facets, with stronger associations of self‐reported health with affective and cognitive well‐being facets and weaker associations with the examined eudaimonic well‐being facets, especially autonomy. These findings complement existing studies on the relationship between self‐reported health and well‐being (e.g., Ryff et al., 2015; Steptoe et al., 2015) by providing a comprehensive overview of the relationships for a broad set of well‐being measures.

### Cross‐lagged effects (H3 and H4)

The third hypothesis stated that deviations from the well‐being trait levels would positively predict subsequent deviations from the hair cortisol trait levels and negatively predict subsequent deviations from the self‐rated health trait levels. The present analyses did not support this hypothesis as none of the examined cross‐lagged effects were significantly different from zero in the expected direction. To contextualize the null findings for hair cortisol, it seems helpful to compare the present study to existing studies that found effects of well‐being on cortisol output. Most of these studies examined how (affective) well‐being affects the acute cortisol output measured in saliva samples and either actively manipulated well‐being through mood induction (e.g., Buchanan et al., 1999) or analyzed pre‐existing differences in well‐being levels (e.g., Polk et al., 2005; Steptoe et al., 2005). In contrast, the present study examined whether naturally occurring within‐person fluctuations in well‐being levels predict the aggregated cortisol output in the subsequent three months measured in hair samples. It could therefore be the case that changes in well‐being primarily affect short‐term cortisol output but that there are no persistent effects on the cortisol system. This explanation challenges the notion that lasting changes in the output of cortisol may be a key bio‐physiological pathway through which higher levels of well‐being manifest themselves as better health. Alternative explanations are that (i) the three‐month period of the present study is too short for changes in well‐being to have persistent effects on cortisol output or (ii) the effects of naturally occurring within‐person variations in well‐being on cortisol output are too small compared to those examined in existing studies. To better understand the temporal progression and magnitude of the effects of changes in well‐being on long‐term cortisol output, more longitudinal research with varying time lags between the measurement (or manipulation) of well‐being and the measurement of cortisol is desperately needed.

In terms of self‐rated health, the analyses analogously indicated that within‐person deviations from the well‐being trait levels did not predict within‐person deviations from self‐rated health trait levels in the subsequent three months. Interestingly, this was the case even though the concurrent correlations between these within‐person deviations were generally positive. This finding suggests that individuals tend to report higher well‐being (compared to the trait levels) when they feel healthier and vice versa. However, elevated well‐being levels do not seem to predict future health assessments.

Lastly, the fourth hypothesis stated that deviations from the hair cortisol trait levels would negatively predict subsequent deviations from the well‐being trait levels, whereas deviations from the self‐rated health trait levels would positively predict subsequent deviations from the well‐being trait levels. However, also H4 was not supported by the analyses as almost all of the cross‐lagged effects were not significantly different from zero in the expected direction. The only exception was that trait level deviations in self‐reported health positively predicted subsequent trait level deviations in life satisfaction within the next three months. Even though this cross‐lagged effect was significantly greater than zero, the size of the cross‐lagged effect was small and indicates that changing self‐reported health by 10 percentage points would result in an 0.46 percentage point increase in life satisfaction when all other variables in the model would be held constant. Moreover, this effect was not significantly different from zero in the two sensitivity analyses, warranting further investigation. Overall, the present study therefore suggests that changes in hair cortisol concentration or self‐rated health do not seem to have an effect on (changes in) well‐being levels in the subsequent three months.

### Limitations and future directions

The present study is based on a large and diverse sample of working‐age individuals and employs a multi‐method approach to comprehensively measure a broad set of well‐being facets, accumulated cortisol output over three months, and self‐rated health. Still, several limitations should be considered when interpreting the study results. First, as discussed above, the present study is based on quarterly measurements. However, longer time intervals might be needed for (changes in) well‐being to have lasting effects on cortisol output (see Schlotz et al., 2008). More research is therefore needed to understand the timeframes in which changes in well‐being can have long‐term effects on cortisol output. Future studies should ideally also measure dehydroepiandrosterone (DHEA), a hormone that has been found to counteract the effects of cortisol and that can also be assessed in hair samples (Buoso et al., 2011). The interplay between cortisol and DHEA may be of particular interest, as it has recently been associated with dispositional optimism in older adults (Zapater‐Fajarí et al., 2024). Second, the primary goal of the data collection was to investigate the effects of unemployment on health and well‐being. Therefore, all examined individuals were initially at high risk of losing their jobs. This initial employment‐related uncertainty and the potential experience of unemployment subsequently was found to have an effect on well‐being and cortisol levels (Lawes et al., 2023; Lawes, Hetschko, Schöb, et al., 2022). While contextual influences such as employment‐related uncertainty, marital problems, and illnesses are captured by the occasion‐specific residual variables, it is not feasible to explicitly model them within the presented analyses. However, the robustness of the study's conclusions was supported by the two sensitivity analyses we presented. These analyses demonstrate that the results remained largely unchanged when (i) observations before and during the COVID‐19 pandemic were analyzed separately and (ii) only continuously employed individuals were examined. Third, while the analyses allow to control for pre‐existing differences in well‐being, health, and cortisol output, there might be time‐varying influences that affect the within‐person changes. Therefore, the reported cross‐lagged effects might be biased and do not necessarily represent causal effects. To effectively probe the causal nature of the effects, the well‐being levels and/or the cortisol output would need to be exogenously manipulated.

## CONCLUSIONS

The present study indicates that hair cortisol levels were generally not associated with the examined affective, cognitive, and eudaimonic well‐being facets, regardless of the level of analysis. Across all examined facets of well‐being, deviations from well‐being trait levels were not associated with subsequent changes in long‐term cortisol output measured in hair samples, challenging the notion that lasting changes in the output of cortisol might be a key bio‐physiological pathway through which higher levels of well‐being manifest themselves as better health. In contrast, self‐reported health was positively correlated with affective, cognitive, and eudaimonic well‐being, albeit to different extents. Overall, these findings underline the importance of longitudinal studies and multi‐methodological methods to investigate the interplay between well‐being and physical health.

## CONFLICT OF INTEREST STATEMENT

The authors declare that they have no known competing financial interests or personal relationships that could have appeared to influence the work reported in this paper.

## ETHICS STATEMENT

The study protocol was approved on December 13, 2017, by the ethics committee of the Department of Education and Psychology at Freie Universität Berlin and informed consent was obtained from all respondents at the start of the entry survey.

## Supporting information


**Table S1.** *Characteristics of Participants at Baseline (Ncohort1 = 449, Ncohort1 = 227).*
**Table S2.** Means, Standard Deviations, and Sample Sizes for the Well‐being and Health Measures at the Five Quarterly Occasions (Q1‐Q5).
**Table S3a.** Model Fit for Different Model Restrictions for the Models with Hair Cortisol
**Table S3b.** Model Fit for Different Model Restrictions for the Models with Self‐Reported Health
**Table S4.** Factor Loadings on the Random Intercept Variables for the Analysis Models
**Table S5a.** Cross‐Lagged Effects of Well‐Being Predicting Health in the Following Three Months
**Table S5b.** Cross‐Lagged Effects of Health Predicting Well‐Being in the Following Three Months.
**Table S6.** Autoregressive Parameters Of the Analysis Models
**Table S7.** Sensitivity Analysis 1: Correlations of Random‐Intercept Variables (Between‐Level) and Occasion‐Specific Residuals (Within‐Level) for Models in Which the Two Cohorts are Analyzed Separately
**Table S8.** Sensitivity Analysis 2: Correlations of Random‐Intercept Variables (Between‐Level) and Occasion‐Specific Residuals (Within‐Level) in Analysis that Only Considers Continuously Employed Individuals
**Figure S1.** Correlations of the Well‐Being and Health Measures over the five Quarterly Occasions
**Figure S2.** Sensitivity Analysis 1: Cross‐Lagged Effects When the Two Cohorts are Analyzed Separately
**Figure S3.**Sensitivity Analysis 2: Cross‐Lagged Effects When Only Individuals are Considered Who Are Continuously Employed

## Data Availability

The data are available for research purposes upon request and can be obtained by contacting the corresponding author.

## Footnotes

^1^
Following a helpful suggestion from an anonymous reviewer, we have rephrased the hypotheses in the article for greater clarity. The content of the originally pre‐registered hypotheses remains unchanged, and their original wording can be found in the pre‐registration: https://osf.io/dg8u7.

^2^
Hair samples were treated as not valid when insufficient hair material was sent in (i.e., less than 3 cm or less than 7.5 g).

^3^
We used multiple regression models to analyze, separately for each quarterly hair collection wave, whether hair color, frequency of hair washing, hair bleaching or dyeing, use of styling products (e.g., gels or sprays), use of styling devices (e.g., straightener), use of hair care products (e.g., conditioner), or use of tanning beds predict hair cortisol concentration (which was Winsorized and log‐transformed). The results can be found in the online repository of this study (https://osf.io/9xbd5/, “RegressionTable_hairConfounds.pdf”) and indicate that none of these factors was consistently related to hair cortisol concentration.
