# Supplementary material for: Longitudinal associations between well‐being, hair cortisol, and self‐reported health
Source: Appl Psychol Health Well Being. 2024 Dec 12;17(1):e12628. doi: 10.1111/aphw.12628 (PMC11638667; doi:10.1111/aphw.12628)
Supplement: Supplementary file 1 — Table S1. Characteristics of Participants at Baseline (Ncohort1 = 449, Ncohort1 = 227). Table S2. Means, Standard Deviations, and Sample Sizes for the Well‐being and Health Measures at the Five Quarterly Occasions (Q1‐Q5). Table S3a. Model Fit for Different Model Restrictions for the Models with Hair Cortisol Table S3b. Model Fit for Different Model Restrictions for the Models with Self‐Reported Health Table S4. Factor Loadings on the Random Intercept Variables for the Analysis Models Table S5a. Cross‐Lagged Effects of Well‐Being Predicting Health in the Following Three Months Table S5b. Cross‐Lagged Effects of Health Predicting Well‐Being in the Following Three Months. Table S6. Autoregressive Parameters Of the Analysis Models Table S7. Sensitivity Analysis 1: Correlations of Random‐Intercept Variables (Between‐Level) and Occasion‐Specific Residuals (Within‐Level) for Models in Which the Two Cohorts are Analyzed Separately Table S8. Sensitivity Analysis 2: Correlations of Random‐Intercept Variables (Between‐Level) and Occasion‐Specific Residuals (Within‐Level) in Analysis that Only Considers Continuously Employed Individuals Figure S1. Correlations of the Well‐Being and Health Measures over the five Quarterly Occasions Figure S2. Sensitivity Analysis 1: Cross‐Lagged Effects When the Two Cohorts are Analyzed Separately Figure S3.Sensitivity Analysis 2: Cross‐Lagged Effects When Only Individuals are Considered Who Are Continuously Employed [file APHW-17-0-s001.pdf]

Supplemental Materials for the following article:

Lawes, M., & Eid, Michael. (2024). Longitudinal associations between well-being, hair cortisol, and self-reported health. *Applied Psychology: Health and Well-Being*.

https://doi.org/10.1111/aphw.12628

Material S1: Questionnaire Items

Self-Rated Health

Source: German Ageing Survey (Engstler et al., 2013)

How do you assess your current health status? [“Wie bewerten Sie Ihren derzeitigen Gesundheitszustand?“]

Scale: disagree completely (1) to agree completely (5)

Satisfaction With Life Scale

Source: Satisfaction With Life Scale (Diener et al., 1985, p. 72; Schumacher, 2003, p. 2)

Below are five statements that you can agree with or disagree with. Please indicate how much you agree with each statement.

Item	Item Wording
1	In most ways my life is close to my ideal. [“In den meisten Bereichen entspricht mein Leben meinen Idealvorstellungen.”]
2	The conditions of my life are excellent. [“Meine Lebensbedingungen sind ausgezeichnet.”]
3	I am satisfied with my life. [„Ich bin mit meinem Leben zufrieden.“]

Scale: do not agree at all (1) to fully agree (7)

Momentary Happiness (ESM)

Source: Multidimensional Mood State Questionnaire (Multidimensionaler Befindlichkeitsfragebogen, for items see Steyer et al., 1994)

Right now I feel happy. [“Im Moment fühle ich mich glücklich.”]
Scale: not at all (1) to very (5)

Adapted Ryff-Scale for Psychological Well-Being

Source: A short version of a German translation of the Ryff-Scale for Psychological Well-Being (Risch et al., 2005; Ryff, 1989) was constructed with an ant algorithm (Schultze, 2017); some negatively worded items were positively rephrased. For items see Schultze (2017, pp. 246–247).

Subscale: Purpose in Life

Item	Item Wording
1	I tend to concentrate on the present because the future almost always brings me problems. [„Ich neige dazu, mich mehr auf die Gegenwart zu konzentrieren, da die Zukunft mir fast immer Probleme bringt.“]

2	I don't have a good sense of what it is I'm trying to accomplish in life. [„Ich weiß nicht so recht, was ich in meinem Leben erreichen möchte.“]
3	I used to set goals for myself, but now it seems like a waste of time to me. [“Früher habe ich mir Ziele gesetzt, aber das kommt mir jetzt wie Zeitverschwendung vor.“]
4	I enjoy making plans for the future and working to make them a reality. [„Ich mache gerne Pläne für die Zukunft und arbeite daraufhin, sie zu verwirklichen.“]

Scale: completely disagree (1) to completely agree (4)

Subscale: Psychological Growth

Item	Item Wording
1	I am interested in activities that will expand my horizons. [„Ich interessiere mich für Aktivitäten, die meinen Horizont erweitern.“]
2	I have really improved as a person over the last years. [“Ich habe mich in den letzten Jahren als Person wirklich weiterentwickelt.“]
3	I gave up trying to make big improvements or changes in my life a long time ago. [“Ich habe schon vor langer Zeit aufgegeben, mein Leben grundsätzlich zu verändern und zu verbessern.“]
4	There's some truth in the saying, “You can't teach an old dog new tricks”. [“Es ist etwas Wahres an dem Spruch: Was Hänschen nicht lernt, lernt Hans nimmermehr.“]

Scale: completely disagree (1) to completely agree (4)

Subscale: Autonomy

Item	Item Wording
1	My decisions are usually influenced by what everyone else is doing. [“Meine Entscheidungen werden normalerweise durch das, was andere machen, beeinflusst.“]
2	Being satisfied with myself is more important to me than what others think of me. [“Mit mir selber zufrieden zu sein, ist mir wichtiger als das, was andere von mir halten.“]
3	I trust my judgment even when it doesn't reflect the convictions of the majority. [“Ich vertraue meinem Urteil, auch wenn es nicht den Überzeugungen der Mehrheit entspricht.“]
4	I judge myself by what I think is important, not by the values of what others think is important. [“Ich beurteile mich selbst nach dem, was ich für wichtig halte, nicht nach den Werten, die für andere gelten.“]

Scale: completely disagree (1) to completely agree (4)

Subscale: Environmental Mastery

Item	Item Wording
1	The demands of everyday life often get me down. [“Die Anforderungen des Alltags entmutigen mich oft.“]
2	I often feel overwhelmed by my responsibilities. [“Ich fühle mich oft von meinen Pflichten erdrückt.“]
3	I manage to organize my time so that I can get everything done that needs to be done. [„Es gelingt mir, meine Zeit so einzuteilen, dass ich alles erledigen kann, was getan werden muss.“]
4	I have difficulty arranging my life in a way that is satisfying to me. [“Es fällt mir schwer mein Leben so zu organisieren, dass es für mich befriedigend ist.“]

Scale: completely disagree (1) to completely agree (4)

Subscale: Positive Relations With Others

Item	Item Wording
1	I often feel lonely because I have few close friends with whom to share my concerns [„Ich fühle mich oft einsam, weil ich nur wenige enge Freunde habe, denen ich meine Sorgen mitteilen kann."]
2	There are a lot of people who will want to listen to me if I have the need to talk. ["Ich habe viele Menschen, die mir zuhören wollen, wenn ich das Bedürfnis habe zu reden."]
3	It seems to me like most other people have more friends than me. ["Mir scheint, dass die meisten anderen Menschen mehr Freunde haben als ich."]
4	I have experienced many warm and trusting relationships with others. ["Ich habe viele warmherzige und vertrauensvolle Beziehungen mit anderen erlebt."]

Scale: completely disagree (1) to completely agree (4)

Subscale: Self-Acceptance

Item	Item Wording
1	In general, I feel confident and positive about myself. [„Im Allgemeinen bin ich selbstbewusst und sehe mich positiv."]
2	I like most parts of my personality ["Ich mag die meisten Seiten meiner Persönlichkeit."]
3	When I compare myself to friends and acquaintances, it makes me feel good about who I am. ["Wenn ich mich mit Freunden und Bekannten vergleiche, habe ich ein gutes Gefühl dabei, so zu sein wie ich bin."]
4	In the past, I have made some mistakes, but all in all, I think most of it has turned out to the best. ["In der Vergangenheit habe ich einige Fehler gemacht, aber ich glaube, alles in allem hat sich das meiste zum Besten gefügt."]

Scale: completely disagree (1) to completely agree (4)

Table S1

Characteristics of Participants at Baseline ($N_{cohort1}$ = 449, $N_{cohort1}$ = 227)

	Cohort 1		Cohort 2	
	N	%	N	%
Gender				
Female	307	68.4	180	65
Male	141	31.4	97	35
Other	1	0.2	0	0
College degree	231	51.8	174	63
Married	202	45.7	121	44
Hair color				
Blond	215	51.8	128	47.2
Brown	169	40.7	126	46.5
Black	10	2.4	5	1.8
Red	20	4.8	12	4.4
Grey/white	1	0.2	0	0
Bleached hair	133	31.7	65	24
Dyed hair	47	10.5	24	8.7

Note. Participants of Cohort 1 were on average 39.64 years old (*SD* = 10.19), had a mean BMI of 25.55 (*SD* = 5.22) and washed their hair an average of 3.81 times a week (*SD* = 2.01). Participants of Cohort 2 were on average 39.09 years old (*SD* = 10.14), had a mean BMI of 25.14 (*SD* = 5.82) and washed their hair an average of 3.55 times a week (*SD* = 1.98).

Table S2

Means, Standard Deviations, and Sample Sizes for the Well-being and Health Measures at the Five Quarterly Occasions (Q1-Q5)

	Q1			Q2			Q3			Q4			Q5		
	M	*SD*	*N*	*M*	*SD*	*N*	*M*	*SD*	*N*	*M*	*SD*	*N*	*M*	*SD*	*N*
Well-being measures															
Momentary happiness	63.38	20.38	657	65.06	16.31	599	64.25	17.22	559	64.13	17.93	521	63.16	17.28	498
Life satisfaction	65.13	18.79	726	64.25	17.11	700	65.28	17.98	644	66.05	18.36	598	65.71	18.3	565
Positive relations with others	65.47	24.63	726	67.07	23.03	697	68.54	23.01	643	69.24	23.78	595	69.1	24.38	564
Autonomy	69.95	17.97	726	70.53	15.67	697	70.87	16.04	643	71.75	16.71	595	71.93	17.11	564
Self-acceptance	74.53	17.51	726	74.27	16.78	697	74.44	17.24	643	74.88	17.68	595	74.83	17.98	564
Psychological growth	78.2	15.77	726	78.9	14.92	697	78.7	16.09	643	78.91	16.59	595	78.7	16.72	564
Sense of purpose	67.23	20.05	725	67.96	17.74	697	67.96	18.89	643	68.38	19.81	595	68.77	19.87	564
Environmental mastery	60.58	21.03	726	60.77	19.94	697	61.17	20.83	643	61.54	21.58	595	60.72	22.56	564
Health measures															
Hair cortisol (log-transformed)	1.61	0.68	687	1.36	0.69	424	1.4	0.74	314	1.43	0.63	268	1.42	0.66	219
Self-rated health	67.67	23.53	726	69.68	19.32	695	70.55	19.17	641	70.24	19.18	594	70.79	18.97	562

Notes. Hair cortisol levels (in pg per mg hair) were winsorized and log-transformed before analysis. The well-being measures and the self-rated health measure were transformed into POMP scores and range from 0–100.

Table S3a

Model Fit for Different Model Restrictions for the Models with Hair Cortisol

		Model Fit						Model Comparison: Likelihood-Ratio-Test		
Facet	Model Type	N_p	χ^2	df	p-value	BIC	RMSEA	$\Delta\chi^2$	Δdf	p-value
Momentary happiness	free	52	29.28	13	0.006	26576.02	0.04 [0.019;0.06]			
Momentary happiness	fixedWithin	31	61	34	0.003	26479.03	0.038 [0.025;0.051]	33.09	21	0.045
**Momentary happiness**	**fixedAll**	**23**	**97.14**	**42**	**<.001**	**26462.94**	**0.046 [0.035;0.057]**	**41.09**	**8**	**<.001**
Life satisfaction	free	52	21.86	13	0.058	28168.49	0.028 [0;0.05]			
**Life satisfaction**	**fixedWithin**	**31**	**53.21**	**34**	**0.019**	**28076.7**	**0.036 [0.023;0.049]**	**31.91**	**21**	**0.06**
Life satisfaction	fixedAll	23	109.03	42	<.001	28087.42	0.054 [0.043;0.064]	67.74	8	<.001
Positive relations with others	free	52	32.81	13	0.002	28597.58	0.04 [0.019;0.06]			
Positive relations with others	fixedWithin	31	90.64	34	<.001	28532.14	0.052 [0.041;0.064]	57.22	21	<.001
**Positive relations with others**	**fixedAll**	**23**	**131.87**	**42**	**<.001**	**28518.14**	**0.057 [0.046;0.067]**	**46.82**	**8**	**<.001**
Autonomy	free	52	24.19	13	0.029	27355.24	0.033 [0.007;0.054]			
Autonomy	fixedWithin	31	66.32	34	<.001	27266.61	0.04 [0.027;0.052]	41.91	21	0.004
**Autonomy**	**fixedAll**	**23**	**109.8**	**42**	**<.001**	**27257.9**	**0.05 [0.039;0.06]**	**47.94**	**8**	**<.001**
Self-acceptance	free*									
**Self-acceptance**	**fixedWithin**	**31**	**58.77**	**34**	**0.005**	**27389.26**	**0.036 [0.022;0.049]**	**-**	**-**	
Self-acceptance	fixedAll	23	129.91	42	<.001	27410.29	0.056 [0.046;0.067]	83.09	8	<.001
Psychological growth	free	52	20.04	13	0.094	26928.82	0.026 [0;0.048]			
**Psychological growth**	**fixedWithin**	**31**	**79.1**	**34**	**<.001**	**26861.74**	**0.048 [0.036;0.06]**	**56.64**	**21**	**<.001**
Psychological growth	fixedAll	23	182.47	42	<.001	26917.97	0.072 [0.062;0.082]	124.47	8	<.001
Sense of purpose	free	52	23.85	13	0.033	28259.86	0.031 [0;0.053]			
**Sense of purpose**	**fixedWithin**	**31**	**56.3**	**34**	**0.009**	**28163.29**	**0.035 [0.021;0.048]**	**33.06**	**21**	**0.046**
Sense of purpose	fixedAll	23	123.82	42	<.001	28180.88	0.055 [0.045;0.066]	81.72	8	<.001
Environmental mastery	free*									
**Environmental mastery**	**fixedWithin**	**31**	**90.63**	**34**	**<.001**	**28580.07**	**0.052 [0.04;0.063]**	**-**	**-**	
Environmental mastery	fixedAll	23	171.58	42	<.001	28608.59	0.067 [0.058;0.078]	94.06	8	<.001

Notes. * negative variances or covariances; ** did not converge; bold models were used for analysis; 90% confidence intervals are presented in square brackets; free: factor loadings and within-person process (i.e., auto-regressive and cross-lagged effects as well as the [co-]variances of the occasion-specific residuals) was not restricted; fixedWithin: within-process was assumed to be constant over time, factor loadings of the random intercept variables were freely estimated. fixedAll: the within-person processes was restricted to be constant over time and the factor loadings of the random intercept variables were fixed to 1.

Table S3b

Model Fit for Different Model Restrictions for the Models with Self-Reported Health

		Model Fit						Model Comparison: Likelihood-Ratio-Test		
Facet	Model Type	N_p	χ^2	df	*p*-value	BIC	RMSEA	$\Delta\chi^2$	Δdf	*p*-value
Momentary happiness	free	52	7.6	13	0.869	48969.31	0 [0;0.024]			
Momentary happiness	fixedWithin	31	44.78	34	0.102	48876.68	0.029 [0.013;0.042]	36.08	21	0.021
**Momentary happiness**	**fixedAll**	**23**	**63.18**	**42**	**0.019**	**48843.21**	**0.032 [0.019;0.044]**	**20.11**	**8**	**0.01**
Life satisfaction	free**									
Life satisfaction	fixedWithin	31	44.15	34	0.114	50351.91	0.032 [0.018;0.045]		-	
**Life satisfaction**	**fixedAll**	**23**	**83.83**	**42**	**<.001**	**50347.28**	**0.046 [0.036;0.057]**	**47.66**	**8**	**<.001**
Positive relations with others	free	52	9.45	13	0.738	51103.89	0 [0;0.028]			
Positive relations with others	fixedWithin	31	79.15	34	<.001	51048.84	0.049 [0.037;0.061]	65.85	21	<.001
**Positive relations with others**	**fixedAll**	23	104.06	**42**	**<.001**	**51019.66**	**0.049 [0.039;0.06]**	**26.58**	**8**	**<.001**
Autonomy	free**									
Autonomy	fixedWithin	31	60.79	34	0.003	49876.79	0.038 [0.025;0.05]		-	
**Autonomy**	**fixedAll**	**23**	**89.01**	**42**	**<.001**	**49853.93**	**0.043 [0.032;0.054]**	**29.67**	**8**	**<.001**
Self-acceptance	free*									
**Self-acceptance**	**fixedWithin**	**31**	**47.69**	**34**	**0.06**	**49823.76**	**0.03 [0.014;0.043]**		**-**	
Self-acceptance	fixedAll	23	101.11	42	<.001	49829.4	0.049 [0.038;0.059]	60.9	8	<.001
Psychological growth	free	52	16.1	13	0.244	49428.71	0.017 [0;0.042]			
**Psychological growth**	**fixedWithin**	**31**	**82.87**	**34**	**<.001**	**49370.89**	**0.05 [0.039;0.062]**	**63.09**	**21**	**<.001**
Psychological growth	fixedAll	23	165.82	42	<.001	49408.24	0.069 [0.059;0.079]	93.48	8	<.001
Sense of purpose	free	52	10.81	13	0.626	50766.02	0 [0;0.032]			
**Sense of purpose**	**fixedWithin**	**31**	**45.5**	**34**	**0.09**	**50669.7**	**0.028 [0.011;0.042]**	**33.48**	**21**	**0.041**
Sense of purpose	fixedAll	23	95.92	42	<.001	50671.54	0.046 [0.036;0.057]	58.51	8	<.001
Environmental mastery	free	52	8.61	13	0.802	51092.78	0 [0;0.025]			
**Environmental mastery**	**fixedWithin**	**31**	**68.5**	**34**	**<.001**	**51020.01**	**0.041 [0.028;0.053]**	**58.63**	**21**	**<.001**
Environmental mastery	fixedAll	23	135.24	42	<.001	51036.3	0.058 [0.048;0.068]	72.82	8	<.001

Notes. * negative variances or covariances; ** did not converge; bold models were used for analysis; 90% confidence intervals are presented in square brackets; free: factor loadings and within-person process (i.e., auto-regressive and cross-lagged effects as well as the [co-]variances of the occasion-specific residuals) was not restricted; fixedWithin: within-process was assumed to be constant over time, factor loadings of the random intercept variables were freely estimated. fixedAll: the within-person processes was restricted to be constant over time and the factor loadings of the random intercept variables were fixed to 1.

Table S4

Factor Loadings on the Random Intercept Variables for the Analysis Models

		Well-Being Indicators					Health Indicators				
Well-Being Facet	Health Measure	Q1	Q2	Q3	Q4	Q5	Q1	Q2	Q3	Q4	Q5
Momentary happiness	Hair cortisol	1 (fixed)	1 (fixed)	1 (fixed)	1 (fixed)	1 (fixed)	1 (fixed)	1 (fixed)	1 (fixed)	1 (fixed)	1 (fixed)
Momentary happiness	Self-rated health	1 (fixed)	1 (fixed)	1 (fixed)	1 (fixed)	1 (fixed)	1 (fixed)	1 (fixed)	1 (fixed)	1 (fixed)	1 (fixed)
Life satisfaction	Hair cortisol	1 (fixed)	1.17 [1.08; 1.26]	1.27 [1.16; 1.38]	1.31 [1.18; 1.43]	1.31 [1.17; 1.44]	1 (fixed)	1.02 [0.86; 1.18]	1.13 [0.95; 1.3]	0.76 [0.6; 0.92]	0.98 [0.83; 1.14]
Life satisfaction	Self-rated health	1 (fixed)	1 (fixed)	1 (fixed)	1 (fixed)	1 (fixed)	1 (fixed)	1 (fixed)	1 (fixed)	1 (fixed)	1 (fixed)
Positive relations with others	Hair cortisol	1 (fixed)	1 (fixed)	1 (fixed)	1 (fixed)	1 (fixed)	1 (fixed)	1 (fixed)	1 (fixed)	1 (fixed)	1 (fixed)
Positive relations with others	Self-rated health	1 (fixed)	1 (fixed)	1 (fixed)	1 (fixed)	1 (fixed)	1 (fixed)	1 (fixed)	1 (fixed)	1 (fixed)	1 (fixed)
Autonomy	Hair cortisol	1 (fixed)	1 (fixed)	1 (fixed)	1 (fixed)	1 (fixed)	1 (fixed)	1 (fixed)	1 (fixed)	1 (fixed)	1 (fixed)
Autonomy	Self-rated health	1 (fixed)	1 (fixed)	1 (fixed)	1 (fixed)	1 (fixed)	1 (fixed)	1 (fixed)	1 (fixed)	1 (fixed)	1 (fixed)
Self-acceptance	Hair cortisol	1 (fixed)	1.2 [1.11; 1.28]	1.26 [1.17; 1.36]	1.29 [1.18; 1.4]	1.31 [1.2; 1.42]	1 (fixed)	1.03 [0.87; 1.19]	1.14 [0.96; 1.31]	0.77 [0.6; 0.93]	0.99 [0.84; 1.15]
Self-acceptance	Self-rated health	1 (fixed)	1.2 [1.11; 1.28]	1.26 [1.16; 1.36]	1.29 [1.18; 1.4]	1.31 [1.2; 1.42]	1 (fixed)	1.01 [0.92; 1.1]	1.05 [0.95; 1.15]	1.03 [0.93; 1.14]	1.03 [0.92; 1.13]
Psychological growth	Hair cortisol	1 (fixed)	1.22 [1.13; 1.32]	1.37 [1.26; 1.48]	1.4 [1.28; 1.52]	1.41 [1.29; 1.53]	1 (fixed)	1.04 [0.88; 1.19]	1.15 [0.98; 1.33]	0.76 [0.6; 0.92]	0.99 [0.83; 1.14]
Psychological growth	Self-rated health	1 (fixed)	1.22 [1.13; 1.31]	1.37 [1.26; 1.48]	1.39 [1.28; 1.51]	1.4 [1.28; 1.52]	1 (fixed)	1.01 [0.92; 1.1]	1.04 [0.94; 1.14]	1.03 [0.92; 1.13]	1.02 [0.91; 1.13]
Sense of purpose	Hair cortisol	1 (fixed)	1.11 [1.03; 1.18]	1.21 [1.12; 1.3]	1.26 [1.16; 1.36]	1.27 [1.17; 1.37]	1 (fixed)	1.02 [0.87; 1.18]	1.13 [0.96; 1.3]	0.75 [0.59; 0.92]	0.99 [0.83; 1.14]
Sense of purpose	Self-rated health	1 (fixed)	1.11 [1.03; 1.18]	1.21 [1.12; 1.3]	1.26 [1.16; 1.36]	1.27 [1.17; 1.37]	1 (fixed)	1.01 [0.92; 1.1]	1.05 [0.95; 1.15]	1.03 [0.93; 1.14]	1.03 [0.92; 1.13]
Environmental mastery	Hair cortisol	1 (fixed)	1.13 [1.06; 1.2]	1.2 [1.13; 1.28]	1.26 [1.17; 1.34]	1.3 [1.21; 1.39]	1 (fixed)	1.01 [0.85; 1.16]	1.11 [0.94; 1.28]	0.75 [0.59; 0.92]	0.98 [0.83; 1.14]
Environmental mastery	Self-rated health	1 (fixed)	1.14 [1.06; 1.21]	1.21 [1.13; 1.28]	1.26 [1.18; 1.34]	1.31 [1.22; 1.4]	1 (fixed)	1.01 [0.92; 1.1]	1.06 [0.96; 1.16]	1.05 [0.94; 1.15]	1.04 [0.93; 1.14]

Note. 95% confidence intervals are presented in square brackets

Table S5a

Cross-Lagged Effects of Well-Being Predicting Health in the Following Three Months

	Hair Cortisol			Self-Reported Health		
	Estimate	90%-CI	*p* (corrected)	Estimate	90%-CI	*p* (corrected)
Momentary happiness	0.001	[-0.001;0.003]	0.325	0.05	[0.007;0.093]	0.19
Life satisfaction	0	[-0.003;0.003]	0.471	0.02	[-0.03;0.07]	0.325
Positive relations with others	-0.003	[-0.006;0]	0.19	0.049	[0.002;0.096]	0.19
Autonomy	-0.004	[-0.007;0]	0.19	0.033	[-0.022;0.088]	0.261
Self-acceptance	0.001	[-0.003;0.005]	0.295	0.089	[0.034;0.143]	0.059
Psychological growth	0.002	[-0.002;0.007]	0.351	0.042	[-0.023;0.106]	0.261
Sense of purpose	0.001	[-0.003;0.004]	0.219	0.018	[-0.034;0.069]	0.325
Environmental mastery	-0.003	[-0.006;0]	0.19	0.03	[-0.02;0.081]	0.261

Note. 95% confidence intervals are presented in square brackets

Table S5b

Cross-Lagged Effects of Health Predicting Well-Being in the Following Three Months

	Hair Cortisol			Self-Reported Health		
	Estimate	90%-CI	*p* (corrected)	Estimate	90%-CI	*p* (corrected)
Momentary happiness	-0.011	[-1.31;1.288]	0.494	0.025	[-0.02;0.071]	0.319
Life satisfaction	0.412	[-0.49;1.314]	0.438	0.046	[0.019;0.074]	0.046
Positive relations with others	0.079	[-0.794;0.953]	0.186	0.029	[-0.003;0.061]	0.186
Autonomy	-0.143	[-0.896;0.61]	0.494	0.018	[-0.005;0.041]	0.213
Self-acceptance	0.889	[0.106;1.671]	0.494	0.023	[0.001;0.046]	0.168
Psychological growth	0.69	[-0.089;1.469]	0.494	0.024	[0.001;0.048]	0.168
Sense of purpose	0.827	[-0.031;1.686]	0.494	0.017	[-0.011;0.044]	0.315
Environmental mastery	1.183	[0.251;2.115]	0.494	0.035	[0.005;0.066]	0.168

Note. 95% confidence intervals are presented in square brackets

Table S6

Autoregressive Parameters Of the Analysis Models

	Hair Cortisol Models		Self-Reported Health Models	
	Well-Being	Hair Cortisol	Well-Being	Self-Reported Health
Momentary happiness	0.16 [0.091;0.229]	0.084 [-0.011;0.18]	0.154 [0.086;0.222]	0.191 [0.134;0.248]
Life satisfaction	0.276 [0.217;0.335]	0.079 [-0.02;0.179]	0.265 [0.207;0.323]	0.194 [0.137;0.251]
Positive relations with others	0.277 [0.22;0.334]	0.084 [-0.014;0.182]	0.275 [0.218;0.332]	0.193 [0.136;0.251]
Autonomy	0.23 [0.183;0.276]	0.083 [-0.013;0.178]	0.231 [0.184;0.278]	0.197 [0.139;0.254]
Self-acceptance	0.237 [0.186;0.289]	0.081 [-0.019;0.18]	0.237 [0.185;0.288]	0.192 [0.136;0.249]
Psychological growth	0.265 [0.207;0.323]	0.078 [-0.021;0.176]	0.263 [0.204;0.322]	0.193 [0.137;0.249]
Sense of purpose	0.218 [0.166;0.27]	0.081 [-0.02;0.183]	0.219 [0.167;0.272]	0.196 [0.139;0.253]
Environmental mastery	0.243 [0.183;0.304]	0.083 [-0.02;0.187]	0.239 [0.179;0.299]	0.194 [0.136;0.251]

Note. 95% confidence intervals are presented in square brackets

Table S7

Sensitivity Analysis 1: Correlations of Random-Intercept Variables (Between-Level) and Occasion-Specific Residuals (Within-Level) for Models in Which the Two Cohorts are Analyzed Separately

	Cohort 1						Cohort 2					
	Hair Cortisol			Self-Reported Health			Hair Cortisol			Self-Reported Health		
	Estimate	90%-CI	*p* (corrected)	Estimate	90%-CI	*p* (corrected)	Estimate	90%-CI	*p* (corrected)	Estimate	90%-CI	*p* (corrected)
Between-Person Level												
Momentary happiness	0.107	[0.002;0.211]	0.493	**0.587**	[0.511;0.662]	< 0.001	-0.074	[-0.22;0.073]	0.234	**0.559**	[0.474;0.644]	< 0.001
Life satisfaction	0.024	[-0.076;0.124]	0.275	**0.676**	[0.61;0.742]	< 0.001	**-0.16**	[-0.283;-0.037]	0.029	**0.633**	[0.549;0.718]	< 0.001
Positive relations with others	0.082	[-0.008;0.172]	0.493	**0.407**	[0.326;0.488]	< 0.001	-0.033	[-0.172;0.106]	0.371	**0.462**	[0.373;0.551]	< 0.001
Autonomy	0.128	[0.042;0.214]	0.493	**0.232**	[0.139;0.324]	< 0.001	0.021	[-0.116;0.159]	0.134	**0.363**	[0.254;0.472]	< 0.001
Self-acceptance	0.102	[0.01;0.194]	0.493	**0.494**	[0.415;0.573]	< 0.001	-0.084	[-0.224;0.057]	0.201	**0.522**	[0.438;0.606]	< 0.001
Psychological growth	-0.049	[-0.148;0.051]	0.306	**0.427**	[0.342;0.512]	< 0.001	-0.125	[-0.263;0.012]	0.107	**0.396**	[0.298;0.495]	< 0.001
Sense of purpose	-0.053	[-0.149;0.043]	0.29	**0.471**	[0.392;0.55]	< 0.001	-0.012	[-0.154;0.129]	0.442	**0.473**	[0.38;0.565]	< 0.001
Environmental mastery	0.086	[-0.005;0.177]	0.493	**0.536**	[0.46;0.611]	< 0.001	0.017	[-0.123;0.157]	0.116	**0.458**	[0.364;0.552]	< 0.001
Within-Person Level												
Momentary happiness	0.066	[-0.016;0.148]	0.464	**0.181**	[0.116;0.247]	< 0.001	0.029	[-0.067;0.126]	0.234	**0.195**	[0.122;0.268]	< 0.001
Life satisfaction	0.087	[0.014;0.161]	0.475	**0.191**	[0.124;0.257]	< 0.001	-0.098	[-0.211;0.016]	0.158	**0.106**	[0.032;0.179]	0.034
Positive relations with others	-0.009	[-0.09;0.072]	0.464	**0.078**	[0.016;0.14]	0.046	-0.044	[-0.163;0.075]	0.291	**0.124**	[0.036;0.213]	0.034
Autonomy	-0.024	[-0.099;0.051]	0.437	0.037	[-0.023;0.097]	0.265	-0.075	[-0.189;0.039]	0.225	0.087	[0.01;0.163]	0.071
Self-acceptance	0.017	[-0.058;0.092]	0.265	**0.172**	[0.113;0.231]	< 0.001	0.019	[-0.085;0.122]	0.208	**0.158**	[0.092;0.224]	< 0.001
Psychological growth	0.079	[-0.007;0.166]	0.464	**0.149**	[0.087;0.211]	< 0.001	0.035	[-0.093;0.162]	0.231	0.099	[0.017;0.181]	0.063
Sense of purpose	-0.009	[-0.087;0.07]	0.464	**0.099**	[0.035;0.164]	0.015	-0.046	[-0.167;0.076]	0.291	0.021	[-0.064;0.106]	0.345
Environmental mastery	-0.046	[-0.125;0.032]	0.265	**0.132**	[0.069;0.194]	< 0.001	0.036	[-0.096;0.169]	0.231	**0.174**	[0.101;0.246]	< 0.001

Notes. The 90%-confidence intervals (CI) were computed since one-sided tests were used to test the correlations against zero at $\alpha = .05$; the p-values correspond to one-sided p-values that were corrected using the Benjamini-Hochberg procedure, estimates with corrected p-values smaller than 0.05 are shown in bold. In all models the within-person process and the factor loadings were fixed to be equal over time.

Table S8

Sensitivity Analysis 2: Correlations of Random-Intercept Variables (Between-Level) and Occasion-Specific Residuals (Within-Level) in Analysis that Only Considers Continuously Employed Individuals

	Hair Cortisol			Self-Reported Health		
	Estimate	90%-CI	*p* (corrected)	Estimate	90%-CI	*p* (corrected)
Between-Person Level						
Momentary happiness	0.098	[-0.059;0.254]	0.397	**0.565**	[0.455;0.675]	< 0.001
Life satisfaction	-0.135	[-0.274;0.004]	0.081	**0.616**	[0.522;0.709]	< 0.001
Positive relations with others	0.016	[-0.117;0.148]	0.099	**0.355**	[0.235;0.474]	< 0.001
Autonomy	0.185	[0.074;0.297]	0.497	**0.284**	[0.173;0.395]	< 0.001
Self-acceptance	0.004	[-0.138;0.145]	0.027	**0.504**	[0.403;0.605]	< 0.001
Psychological growth	-0.122	[-0.266;0.021]	0.099	**0.4**	[0.282;0.517]	< 0.001
Sense of purpose	**-0.18**	[-0.319;-0.04]	0.027	**0.438**	[0.339;0.536]	< 0.001
Environmental mastery	0.112	[-0.019;0.242]	0.448	**0.478**	[0.378;0.578]	< 0.001
Within-Person Level						
Momentary happiness	-0.024	[-0.117;0.069]	0.385	**0.225**	[0.153;0.296]	< 0.001
Life satisfaction	0.017	[-0.072;0.105]	0.193	**0.192**	[0.102;0.281]	0.001
Positive relations with others	0.057	[-0.042;0.155]	0.385	0.096	[0.019;0.173]	0.055
Autonomy	-0.031	[-0.123;0.061]	0.385	0.051	[-0.017;0.12]	0.193
Self-acceptance	-0.021	[-0.094;0.053]	0.385	**0.147**	[0.072;0.223]	0.003
Psychological growth	0.128	[0.027;0.229]	0.482	0.109	[0.023;0.195]	0.055
Sense of purpose	-0.015	[-0.099;0.069]	0.413	0.082	[-0.003;0.167]	0.113
Environmental mastery	-0.092	[-0.18;-0.005]	0.094	**0.2**	[0.121;0.279]	< 0.001

Notes. The 90%-confidence intervals (CI) were computed since one-sided tests were used to test the correlations against zero at $\alpha = .05$; the p-values correspond to one-sided p-values that were corrected using the Benjamini-Hochberg procedure, estimates with corrected p-values smaller than 0.05 are shown in bold. In all models the within-person process and the factor loadings were fixed to be equal over time.

Figure S1

Correlations of the Well-Being and Health Measures over the five Quarterly Occasions

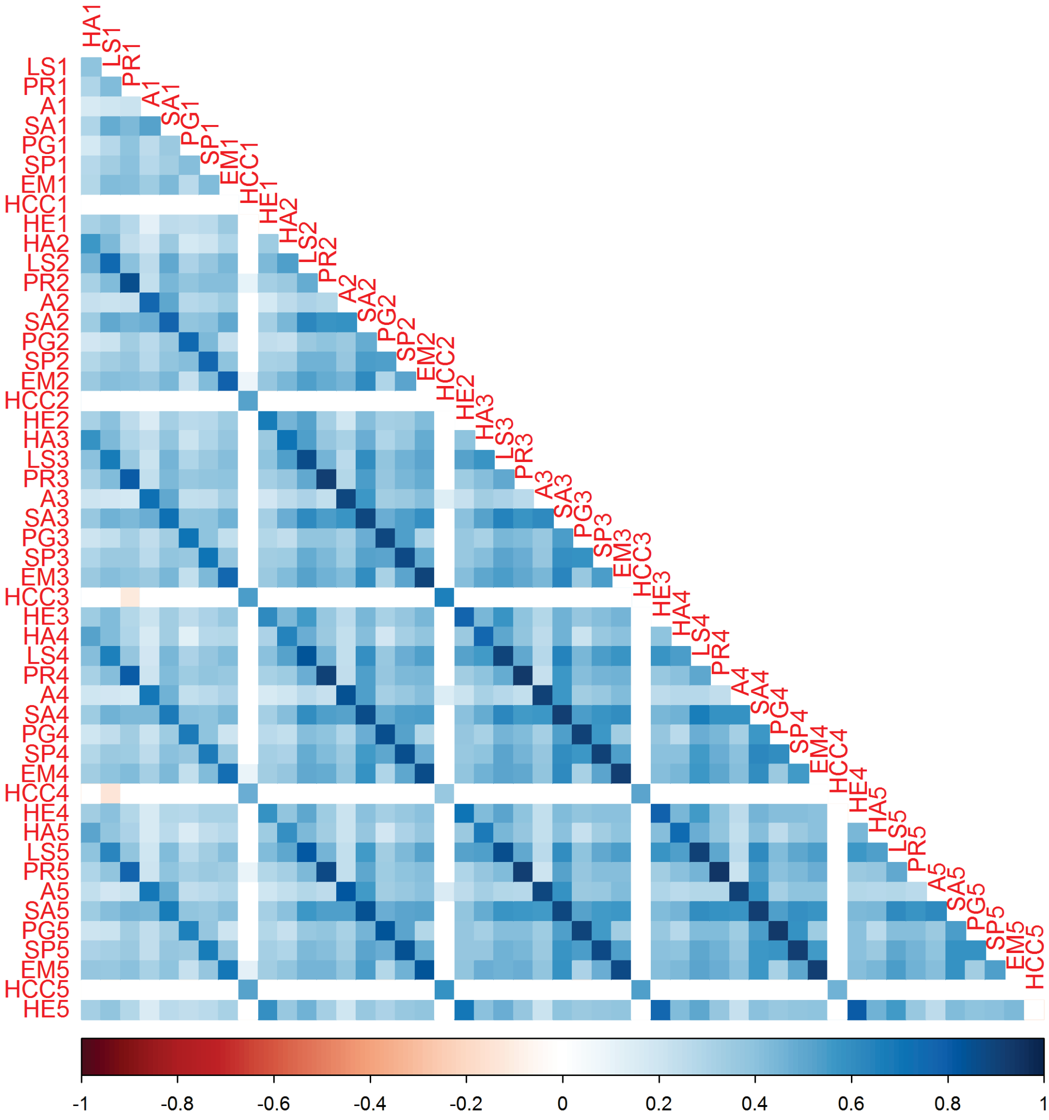

Note. The cells of the correlation matrix correspond to the bi-variate pearson product correlations. Only correlations that were significantly different from zero (α < .05) are colored. The variable names correspond to appreviations of the well-being and health measures (defined below) and the numbers 1-5 indicating the five quarterly occasions. The well-being and health measures are appreviatied as HA: Momentary happiness, LS: Life satisfaction, PR: Positive relations with others, A: Autonomy, SA: Self-acceptance, PG: Psychological growth, SP: Sense of purpose, EM: Environmental mastery, HCC: Hair cortisol (log-transformed), HE: Self-rated health

Figure S2

Sensitivity Analysis 1: Cross-Lagged Effects When the Two Cohorts are Analyzed Separately

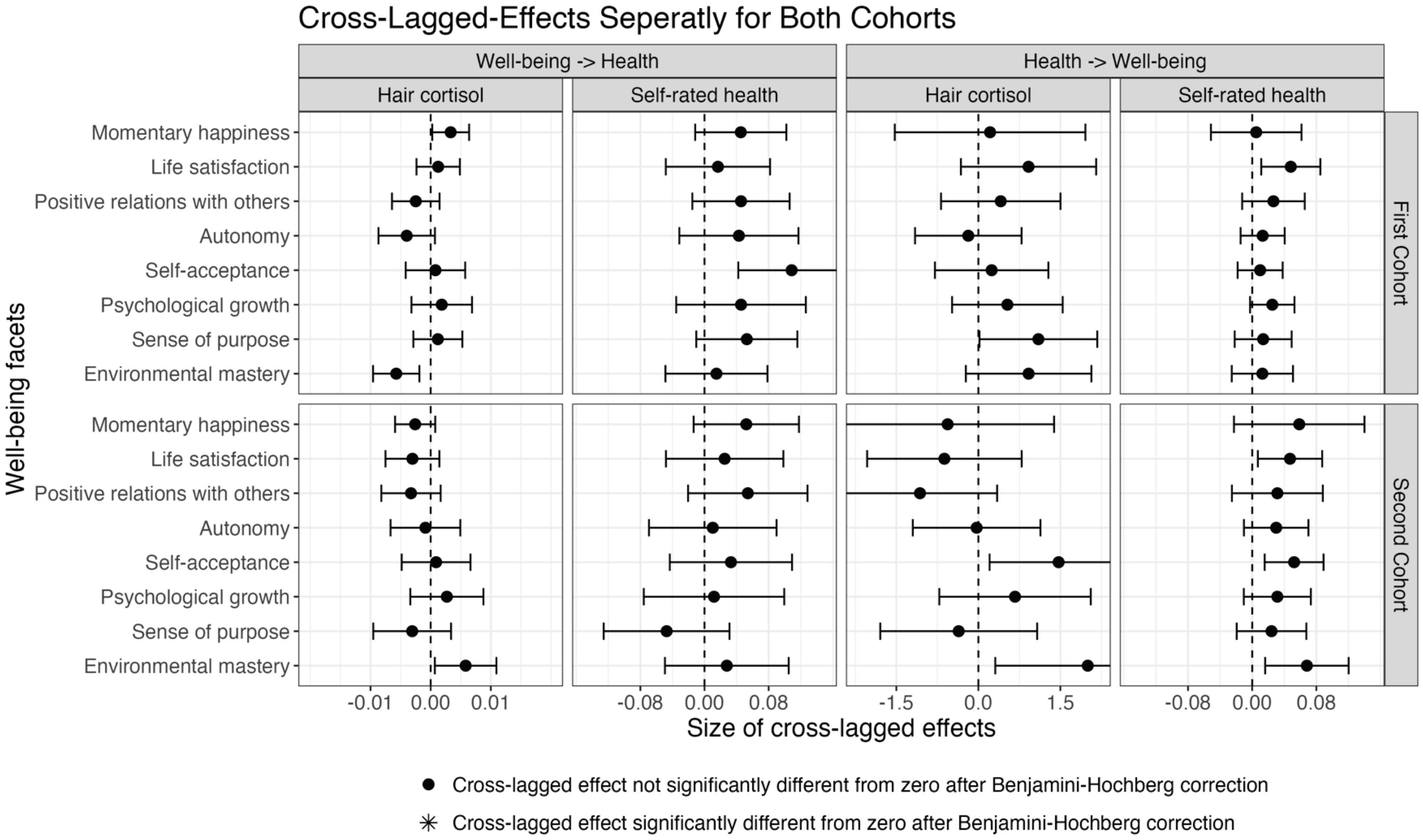

Note. The points depict the cross-lagged effects, the whiskers denote the 90%-confidence interval (i.e., one-sided tests). Note that the scaling of the x-axis differs between the plots, this is due to the different metric in which the variables were analyzed. The first rows depict to the effects in the first cohort, the later rows depict the effects in the second cohort.

Figure S3

Sensitivity Analysis 2: Cross-Lagged Effects When Only Individuals are Considered Who Are Continuously Employed

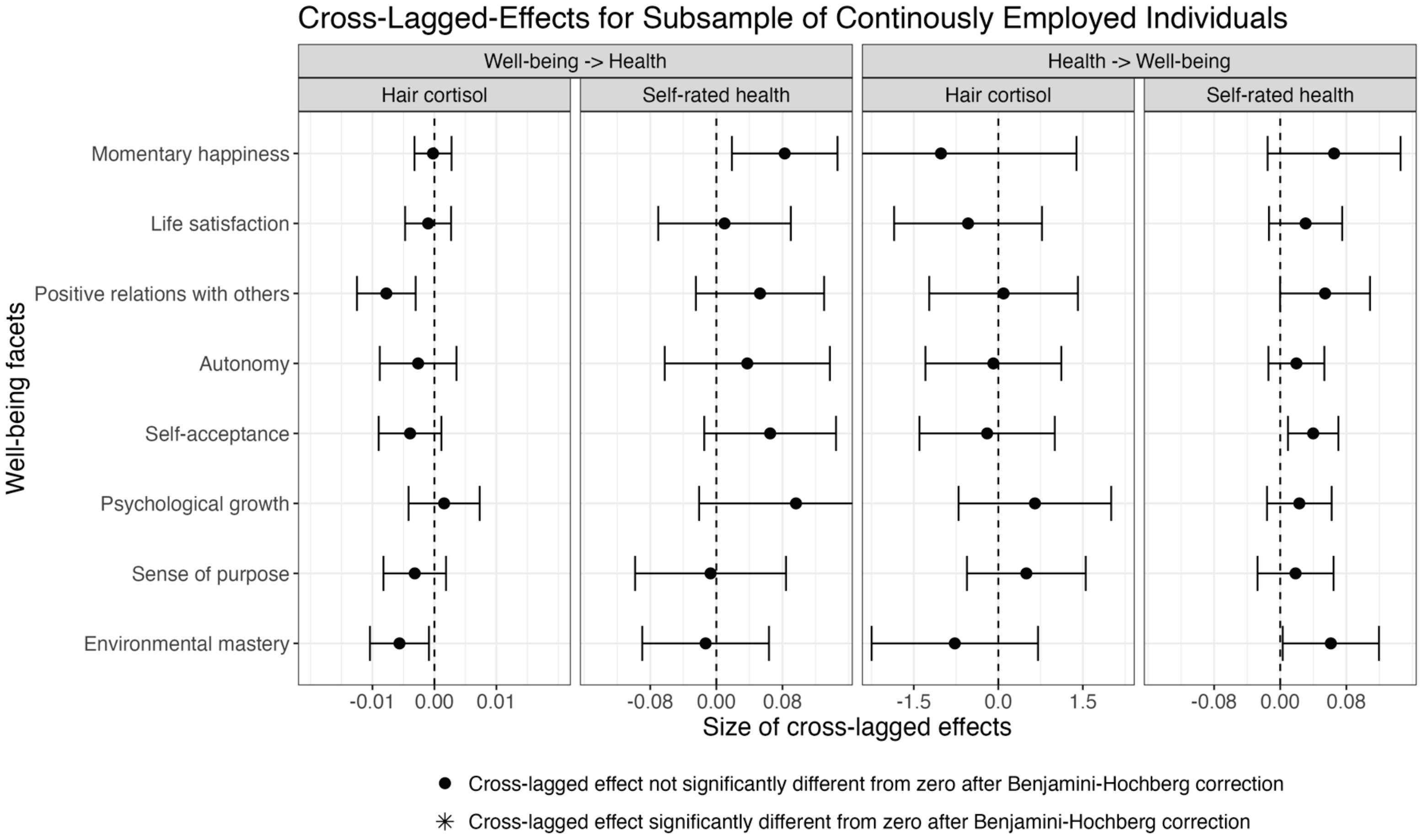

Note. The points depict the cross-lagged effects, the whiskers denote the 90%-confidence interval (i.e., one-sided tests). Note that the scaling of the x-axis differs between the plots, this is due to the different metric in which the variables were analyzed.
